# Cavy lifespan: survival analysis and lifetables for the pet guinea pig (*Cavia porcellus*, L.) in Britain

**DOI:** 10.7717/peerj.19702

**Published:** 2025-07-18

**Authors:** Fernando Mata

**Affiliations:** Center for Research and Development in Agrifood Systems and Sustainability, Instituto Politécnico de Viana do Castelo, Viana do Castelo, Portugal

**Keywords:** Guinea pig, Cavy, *Cavia porcellus*, Survival analysis, Lifetable, Age at death, Lifespan, Pet, Britain

## Abstract

In Britain, an estimated number of 700,000 guinea pigs are currently kept as pets. Despite their widespread presence, research on pet guinea pig survivability remains limited. While demographic studies exist, they primarily focus on morbidity rather than lifespan analysis. This study aims to fill this gap by investigating factors influencing pet guinea pig longevity and constructing life tables, contributing valuable insights into their survival patterns and potential improvements in care practices. The study was conducted using publicly available data from the VetCompass™ UK Program, including 675 guinea pigs receiving veterinary care in the UK in 2019. The survival analysis was conducted using Kaplan–Meier models, with lifespan differences tested via the Log-Rank test. Key predictors included sex, neutering status, and breed classification.

The results of the study indicate an average lifespan of the British pet guinea pigs is 4.022 (3.875; 4,170) years; a median survival time of 4.025 95% confidence interval (3.830; 4,290) years; and an interquartile range of (2.563; 5447) years. Unlike other companion animals, no significant differences in longevity were found between sexes, neutering status, or breed (log-rank Mantel-Cox *χ*2 *p* > 0.05), suggesting that these factors may play a less critical role in guinea pigs. The findings challenge common assumptions about lifespan determinants in domesticated species. The study also highlights low neutering rates, likely influenced by concerns over surgical risks. Additionally, breed classification remains inconsistent, indicating a possible lack of standardised identification among owners and veterinarians. This study is limited by potential selection bias, lack of environmental and husbandry data, few neutered animals, unclear breeding backgrounds, unaccounted cohort effects, and no geographical analysis of care variation across different regions of Britain.

## Introduction

The guinea pig (*Cavia porcellus*, L.) boasts a rich human relationship history that spans continents and centuries. Originating in the Andean highlands of South America, archaeological evidence suggests that Indigenous people of present-day Peru, Bolivia, Ecuador, and Colombia domesticated these rodents as early as 5000 BC (Morales, 1994). In these cultures, guinea pigs served as a vital food source and held significant roles in religious ceremonies and traditional healing practices (deFrance, 2021). This was, however, just the first step in Guinea pig domestication of wild species into the ancient Criollo (Creole) South American breed (Spotorno et al., 2006).

The 16th century marked the introduction of guinea pigs to Europe, primarily through Spanish explorers and traders. By the late 1500s, guinea pigs had reached Spain and gradually spread across the European continent (Pigière et al., 2012). Archaeological evidence suggests that by 1574-5 the guinea pig was already present in Britain (Pigière et al., 2012). Their docile nature and unique appearance made them desirable pets among European elites, including British royalty. Queen Elizabeth I is noted as one of the early enthusiasts, keeping guinea pigs as exotic companions (Grigson, 2016). This royal endorsement elevated the guinea pig’s status, making it a fashionable pet among the European aristocracy (Van Dijk & Silkens, 2012).

As interest in guinea pigs grew, European breeders began selective breeding programs to develop distinct breeds characterised by various coat colours, textures, and patterns. This aspect marks the second step in guinea pig domestication (Spotorno et al., 2006). Breeding for meat is practiced in many countries, especially across South America, Asia, and Africa (Sánchez-Macías et al., 2018). The development of meat traits gave rise to the third step in the domestication process of the guinea pig (Spotorno et al., 2006), with many meat-specialised large breeds developing, such as the Andean, Inti, Peru, and Mantaro (Pinchao-Pinchao et al., 2024).

The Victorian era solidified the guinea pig’s role as a beloved household pet in Britain. Their gentle temperament, ease of care, and suitability for small living spaces made them especially popular among children (Strange, 2021). The formation of guinea pig clubs and societies further contributed to their widespread appeal, promoting responsible ownership and breeding practices (Amato, 2008). The National Cavy Club (NCC) was established in 1888 (Neesam, 2025). Throughout the 20th century, guinea pigs maintained their status as ideal family pets, with educational institutions often incorporating them into programs to teach children about animal care and responsibility (O’Haire et al., 2013; Ormerod, 2022). Today, guinea pigs continue to be among the most popular pets in Britain, with a thriving community of breeders, enthusiasts, and owners that congregate under the NCC, but also the more recently established (1977) British Cavy Council (Neesam, 2020a). In 2024, the estimated number of pet guinea pigs in Britain was around 700,000, with approximately 1.2% of households having at least one (UK Pet Food, 2025).

Advancements in pet care have improved guinea pigs’ well-being, and recent studies have shown that lifespan has increased across many pet species (Serpell, 2018). Factors such as sex, breed or neutering status have been identified, individually or together, as affecting survivability in domestic species such as dogs (Mata & Mata, 2023), cats (Mata, 2025), cattle (Kirkpatrick, 2017), sheep (Málková et al., 2020), chicken (Mata & Mwakifuna, 2012), and Japanese quail (Khan et al., 2010).

VetCompass™ (Veterinary Companion Animal Surveillance System) is a UK-based research program led by the Royal Veterinary College, University of London. It collects and analyses anonymised clinical data from veterinary practices nationwide to study companion animal health. By monitoring diseases, treatments, and outcomes, the VetCompass helps improve animal welfare, guide veterinary practices, and inform public understanding. Its large, real-world database supports evidence-based research to advance veterinary care (VetCompass, 2025).

Studies on survival analysis of the pet guinea pig were never produced before. Demographic studies were conducted in Britain, however, focusing primarily on morbidity, rather than analysis of survivability (O’Neill et al., 2024). The authors found that of the deaths where a cause was documented, the leading causes were anorexia (13.87%), collapse (9.81%), and peri-anaesthetic complications (3.38%). Neoplasia was the only category of cause of death with a significant difference between sexes, with males having 0.44 times the odds of dying from it compared to females. The authors have also found that the most frequently observed conditions were overgrown nails (26.55%), dermatophytosis (6.02%), and corneal ulceration (4.99%). Of the thirty most prevalent disorders, females were more likely to be affected by three, while males showed a higher tendency for five. Dermatophytosis had the youngest average age of affected animals at 1.11 years, whereas weight loss was associated with the oldest average age at 4.64 years. This study aims to investigate survivability and construct life tables for the British pet guinea pig.

## Materials & Methods

The dataset utilised in the present study is publicly available under the Creative Commons Attribution 4.0 license (CC BY 4.0) and was sourced from the Figshare’s repository of the Royal Veterinary College, University of London (O’Neill, 2024). The sample includes all the guinea pigs receiving primary care in UK veterinary clinics enrolled in the VetCompass™ Program during the year 2019. The surveyed guinea pigs had at least one electronic patient entry recorded in this year (O’Neill et al., 2024). The dataset included variables such as ‘neutering status’ (classified as ‘entire’ or ‘neutered/spayed’), ‘guinea pig breed’ (‘domestic’, and ‘pure breeds’), ‘age at death’ in years, and ‘sex’ (male or female).

Originally, the mortality dataset contained *N* = 758 records. However, after data cleaning by removing unknown birth date entries, the sample analysed was reduced to *N* = 675 records. Furthermore, to analyse differences between sex, breeds, and neutering status, the sample was reduced as some entries stated ‘unknown’ for the characteristic. There are no censored entries (such as known age at the end of study without death, or lost to follow-up). The details on the distribution of observations across categories, after removal of unrecorded/unknown, can be found in Table 1. As can be observed, the number of neutered/spayed animals is minimal. The number of purebred pet guinea pigs is also vastly outnumbered by outbred pet domestic guinea pigs.

**Table 1:** Number of entries in the survival analysis of the British pet guinea pigs per level of the factor considered, after removal of unrecorded/unknown.

Sex	N^er^	Breeding status	N^er^	Breed	N^er^	Sex & breeding status	N^er^
Female	321	Neutered/spayed	17	Domestic	627	Female spayed	5
Male	319	Entire	623	Pure breed	47	Male neutered	12
Total	640	Total	640	Total	674	Female entire	316
						Male entire	307
						Total	640

Kaplan–Meier models were adjusted to the data to analyse the survival outcomes, and the lifespan differences were tested using the log-rank (Mantel-Cox) test. A survival analysis plot and a lifetable were produced. The event was defined as ‘age at death’ (in years). The models included ‘breeding status’ (‘entire’ or ‘neutered/spayed’), ‘sex’ (‘male’, ‘female’), the combination ‘sex/neutered status’ (‘entire female,’ ‘spayed female,’ ‘neutered male,’ and ‘entire male’), and ‘breed’ (‘pure’, and ‘outbred’) as key predictors. The purebred group included guinea pigs from the following breeds: Abyssinian, Alpaca, Coronet, Crested, Dutch, Himalayan, Peruvian, Rex, Silkie, and Teddy. The outbred group included Domestic and Domestic Short Hair guinea pigs.

Descriptive statistics and data cleaning were executed in a spreadsheet (Microsoft Excel for Microsoft 365 MSO, version 2204 Build 16.0.15128.20240, 64-bit). The Kaplan–Meier models and the life tables were executed using IBM^®^ SPSS^®^ Statistics, Version 29.0.2.0 (20). A significance level of *p* < 0.05 was applied to all statistical tests.

## Results

The mean lifespan of the British guinea pigs in the analysed dataset is 4.022 years (4 years and 8 days) with a 95% confidence interval (CI) (3.875–4.170) years; the median survival time is 4.025 years with a 95% CI [3.830–4.219] years and interquartile range (IQR) of (2.563, 5.443) years.

**Figure 1:**
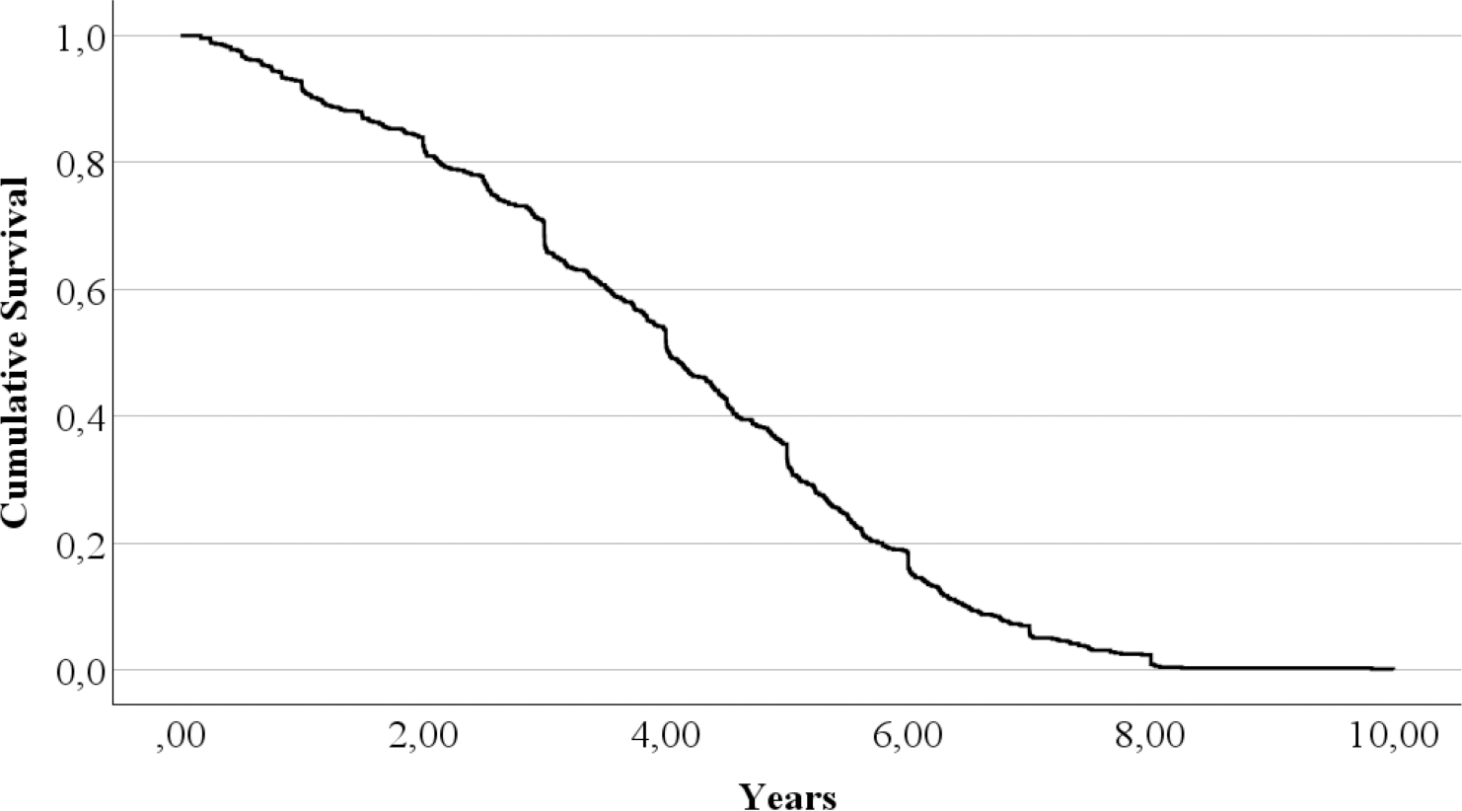
The British pet guinea pig survival curve.

The full statistics, including minimum and maximum, can be found in Table 2. The survival curve is represented in Fig. 1.

The Kaplan–Meier tests applied to the factors ‘sex’, ‘neutering status’, and ‘breed’ were found not significant (*p* > 0.05). Therefore, different lifespans were not found between the groups ‘males’ and ‘females’,’ neutered/spayed’ and ‘entire’, ‘domestic’ outbred and ‘pure breed’, and ‘male entire’, ‘female entire’, ‘male neutered’, and female spayed’ of guinea pigs. The full statistics of the tests, including the Log Rank Mantel-Cox *χ*^2^ values, can be found in Table 3. The overall pet guinea pig life table is presented in Table 4. The guinea pig life table summarises the mortality and survival patterns of the studied guinea pig population over time. It provides detailed information on the probability of death at each age or age group, as well as the expected number of years remaining for individuals at different ages.

**Table 2:** Minimum, mean, median, 95% confidence intervals, interquartile range, and maximum lifespan of the sampled British pet guinea pigs in the present study. Data provided in years.

Mean	Minimum	Standard error	95% Confidence interval		Maximum
			Lower	Upper	
4.022	0.17	0.075	3.875	4.170	10.0

Median	Minimum	Standard error	95% Confidence interval		Maximum
			Lower	Upper	
4.025	0.17	0.099	3.830	4.29	10.0

Median		Minimum	Interquartile Range		Maximum
			25%	75%	
4.025		0.17	2.563	5.447	10.0

**Table 3:** Kaplan–Meier statistics observed while testing significant differences in lifespan, between the levels of the factors indicated, in the British guinea pigs.

Factors	Log Rank Mantel-Cox *χ*2	Degrees of freedom	*P*-value
Sex	0.057	1	0.812
Neutering Status	0.668	1	0.414
Breed	0.102	1	0.750
Sex × Neutering status	4.791	3	0.188

**Table 4:** Lifetable for the British pet guinea pig.

Interval start time	No entering interval	No withdrawing during interval	No exposed to risk	No of terminal events	Proportion terminating	Proportion surviving	Cumulative proportion SEI	SE of cumulative proportion SEI	Probability density	SE of probability density	Hazard rate	SE of hazard rate
0.0	674	0	674	17	0.03	0.97	0.97	0.01	0.050	0.012	0.05	0.01
0.5	657	0	657	38	0.06	0.94	0.92	0.01	0.113	0.018	0.12	0.02
1.0	619	0	619	30	0.05	0.95	0.87	0.01	0.089	0.016	0.10	0.02
1.5	589	0	589	32	0.05	0.95	0.83	0.01	0.095	0.016	0.11	0.02
2.0	557	0	557	37	0.07	0.93	0.77	0.02	0.110	0.018	0.14	0.02
2.5	520	0	520	59	0.11	0.89	0.68	0.02	0.175	0.022	0.24	0.03
3.0	461	0	461	53	0.11	0.89	0.61	0.02	0.157	0.021	0.24	0.03
3.5	408	0	408	47	0.12	0.88	0.54	0.02	0.139	0.020	0.24	0.04
4.0	361	0	361	73	0.20	0.80	0.43	0.02	0.217	0.024	0.45	0.05
4.5	288	0	288	63	0.22	0.78	0.33	0.02	0.187	0.022	0.49	0.06
5.0	225	0	225	61	0.27	0.73	0.24	0.02	0.181	0.022	0.63	0.08
5.5	164	0	164	56	0.34	0.66	0.16	0.01	0.166	0.021	0.82	0.11
6.0	108	0	108	41	0.38	0.62	0.10	0.01	0.122	0.018	0.94	0.14
6.5	67	0	67	26	0.39	0.61	0.06	0.01	0.077	0.015	0.96	0.18
7.0	41	0	41	19	0.46	0.54	0.03	0.01	0.056	0.013	10.21	0.26
7.5	22	0	22	6	0.27	0.73	0.02	0.01	0.018	0.007	0.63	0.25
8.0	16	0	16	14	0.88	0.13	0.00	0.00	0.042	0.011	30.11	0.52
8.5	2	0	2	0	0.00	10.00	0.00	0.00	0.000	0.000	0.00	0.00
9.0	2	0	2	0	0.00	10.00	0.00	0.00	0.000	0.000	0.00	0.00
9.5	2	0	2	2	1.00	0.00	0.00	0.00	0.006	0.004	40.00	0.00

**Notes.**

No number SE standard Error SEI standard error Interval

## Discussion

The lifespan of the guinea pig in the wild is reported to be around 3 years, however, many do not pass their first year (Eisenberg, 1989). The survivability of the pet guinea pig has never been studied before with real data, nor have life tables been produced. It is therefore difficult to discuss the results of the present study by comparison with those of other authors. The findings indicate that the mean lifespan of British pet guinea pigs is 4.022 years, with a 95% CI [3.875–4,170]; and a median survival time of 4.025 years, with a 95% CI [3.830–4,290], and an IQR of (2.563; 5447). These estimates align with previous anecdotal reports on guinea pig longevity. Fawcett (2011) refers to four to nine years, and five to seven if kept indoors; Pignon & Mayer (2020) refer to four to five years for the pet guinea pig lifespan (up to eight years); Kaufmann (2004), estimates up to six years; and Berg (1943) comments that guinea pigs live as many months as humans live years, which would lead to approximately 7.67 years considering a human lifespan of 80 years. Anecdotal evidence refers to owners reporting a 12 to 15 years maximum lifespan (Donnelly & Brown, 2004).

In a real data study conducted in the United Kingdom (Harrup & Rooney, 2020) questionnaires were distributed to 4,719 owners, resulting in 3,390 answers about the lifespan of their most recently owned pet guinea pig. This led to an estimated mean lifespan of 4.1 years, with a standard deviation of 3.1 years. This leads to a calculated 95% CI for the mean of (3.996; 4.204), which is in complete agreement with the results of the present study. These authors have also reported lifespans up to 13 years, and 5.2% of the guinea pigs living above nine years.

Companion animals now benefit from enhanced nutritional formulations, preventative healthcare, and better husbandry practices, all of which contribute to longevity (Wolf, Lloyd & Black, 2008). Over the past two decades, advancements in veterinary medicine, improved pet care, and greater awareness of animal welfare have led to increased lifespans in many domesticated species (Cozzi et al., 2017).

Geriatric care for guinea pigs is an emerging field that emphasises monitoring for age-related conditions such as arthritis, dental disease, and metabolic disorders (Jenkins, 2010). As more pet owners recognize the importance of proactive care, guinea pigs may benefit from the broader trends in companion animal longevity observed over the past decades. With appropriate care, some guinea pigs can live beyond the typical lifespan (Bays, 2020).

A key finding of this study is the lack of statistically significant differences in lifespan based on sex, neutering status, or breed. Unlike other domestic species, such as dogs (Mata & Mata, 2023), and cats (Mata, 2025), where these factors have been shown to significantly influence survival, guinea pigs appear to have relatively uniform longevity across these categories.

No significant differences were found in the survivability of male and female pet guinea pigs. This differs from patterns seen in other species, especially mammals, where females are typically associated with greater longevity. Research on 42 mammalian species spanning eight orders suggests that a prolonged lifespan is common among female mammals (Cohen, 2004).

A possible explanation for the results obtained may be related to the late breeding. In the wild, sows are ready for mating 4 weeks after birth and have perennial oestrus, therefore giving birth several times in a year (Eisenberg, 1989). Pet guinea pig owners may want to wait longer before allowing reproduction. If the sows remain virgin for a long period, their pelvic symphysis fuses, creating serious problems during labour. Richardson (2009) recommends waiting no more than four to five months before putting a sow to a boar to avoid pelvic fusion, and should reproduction be desired. This problem is overcome in multiparous sows as the pelvic bones’ junction remains fibrous after the first parturition, allowing enlargement of the birth canal (Ortega et al., 2003). Caesarean is the alternative in dystocia caused by failure of enlargement of the birth canal; however, the death rate is much higher in guinea pigs by comparison with other pets. The risk of death from anaesthesia or sedation in guinea pigs in the UK is reported at 5.13%, plus 5.29% euthanised, which is significantly higher than that observed for dogs (0.29% and 1.53%), cats (0.31% and 1.65%), rabbits (1.78% and 2.61%), and ferrets (0.67% and 2.0%) (Brodbelt, 2006).

Another possible explanation is that female guinea pigs are prone to developing ovarian cysts, especially after 1.5 years of age. These fluid-filled sacs can grow large, causing pain, hormonal imbalances, hair loss, and decreased appetite. Without treatment, cysts can lead to serious complications and reduced quality of life, and lifespan (Sadar & Gleeson, 2025).

The non-significance in neutering status contrasts with findings in other species, where neutering has often been associated with increased lifespan due to reduced reproductive-related health risks (Serpell, 2018). However, the small number of neutered/spayed individuals in the dataset may have limited the statistical power to detect any potential effects.

In the present study, only 2.66% of the guinea pigs were recorded as neutered, with males being significantly more likely to undergo the procedure than females (3.66 *vs* 1.56%). This figure is notably lower than the 10.9% neutering rate found in a previous study also conducted in the UK using an owner’s survey (Harrup & Rooney, 2020). In terms of proportions between sex, these authors have also identified a higher likelihood of males being neutered compared to females.

The low neutering rate in guinea pigs may be linked to concerns over anaesthetic risks referred to before. The difference in neutering rates between male and female guinea pigs is likely because neutering males is a less complex surgical procedure than spaying females (Rozanska et al., 2016). Additionally, when housing arrangements involve a single male living with multiple females, neutering the male rather than spaying all the females is the more practical option. Harrup & Rooney (2020) report that 78.6% of the pet guinea pigs in the UK are housed with conspecifics. Guinea pigs are highly social animals, and it is generally recommended that they be kept in same-sex pairs, female groups, or mixed-sex groups where neutering may be managed conveniently (Harrup & Rooney, 2020).

The lack of significant lifespan differences between purebred and domestic outbred guinea pigs suggests that genetic factors related to breed type may not be major determinants of longevity in this species. This is in contrast to findings in other companion animals, such as dogs (Mata & Mata, 2023), and cats (Mata, 2025), where breed-related morphological traits have been linked to distinct lifespan patterns. One possible explanation for this finding is that selective breeding for specific traits in guinea pigs has not resulted in the same degree of health trade-offs observed in other species, such as the brachycephalic airway syndrome in certain dog (Krainer & Dupré, 2022), and cat and rabbit (Campbell, 2024) breeds. Historically, guinea pig breed differentiation has been based primarily on variations in coat length, texture, structure, and colour rather than dramatic body shape modifications (Neesam, 2020b). As a result, the breeding component may not hold clinical relevance for guinea pigs when compared to other pets. Also, a study (Williams & Sullivan, 2010) found that outbred pet guinea pigs exhibited a higher occurrence of ocular lesions when compared to pure breeds, challenging the inbreeding depression assumptions.

In the dataset used in this study, 93.0% of the guinea pigs were classified as domestic, and only 7% as pure breeds. This may suggest that most guinea pigs in Britain do not belong to a distinct pure breed. In addition, this could indicate that owners and veterinary professionals either have limited knowledge of guinea pig breeds or place less importance on breed classification compared to other household pets, such as dogs. Therefore, the ‘domestic’ naming may be given to unknown breeds or crosses, rather than outbred guinea pigs. In their survey, Harrup & Rooney (2020) refer to 19% ‘cross-bred’, and 39.8% ‘unknown’. It remains unclear whether the remaining 41.2% named as ‘pure breed’ contain the domestic outbred, or if these are considered under the ‘unknown’. This uncertainty aligns with previous retrospective studies, which either failed to document guinea pig breeds (Bertram, Müller & Klopfleisch, 2018; Minarikova et al., 2015) or reported them inconsistently (Laik-Schandelmaier et al., 2017; White et al., 2016).

This study has several limitations. The dataset used was restricted to guinea pigs receiving veterinary care within the VetCompass™ program, which may introduce selection bias by excluding animals that did not receive veterinary attention. Additionally, the dataset does not account for potential environmental and husbandry-related factors, such as diet, housing conditions, and owner experience, which may play a role in individual longevity. Furthermore, the relatively small number of neutered animals and an apparent confusion in the identification of breeding backgrounds may also have contributed to a bias in results relating neutering status and breeds. The study did not account for potential cohort effects, where guinea pigs born in different years may have faced varied husbandry trends, nor did it analyse geographical variation, possibly overlooking regional care differences.

Future research should aim to address these limitations by incorporating larger, more diverse samples and exploring additional factors that could influence guinea pig lifespan. Longitudinal studies that track guinea pigs from birth to death would be particularly valuable in identifying key determinants of longevity.

## Conclusions

The present study provides the first real-data analysis of pet guinea pig lifespan, confirming a mean of approximately four years. Unlike other companion animals, no significant differences were found in survival based on sex, neutering status, or breed. This contrasts with patterns observed in other mammals, where females often live longer, neutering typically extends lifespan, and breed-specific traits influence longevity. Possible explanations include the effects of late breeding, which may mitigate female longevity advantages, and the relatively minor role of selective breeding in guinea pigs compared to other species. The study also highlights the low neutering rates in pet guinea pigs, likely due to the higher anaesthetic risks associated with these procedures. Breed classification in guinea pigs remains inconsistent, potentially reflecting limited owner knowledge or a lower emphasis on breed distinction in this species.

##  Supplemental Information

10.7717/peerj.19702/supp-1 Supplemental Information 1 Categorical variables code

## Additional Information and Declarations

### Data Availability

The following information was supplied regarding data availability: The data is available at figshare: O’Neill, Dan (2024). Demography, commonly diagnosed disorders and mortality of guinea pigs under primary veterinary care in the UK in 2019—A VetCompass Study. figshare. Dataset. https://doi.org/10.6084/m9.figshare.24936924.v1.
