# Supplementary material for: Cavy lifespan: survival analysis and lifetables for the pet guinea pig (Cavia porcellus, L.) in Britain
Source: PeerJ. 2025 Jul 18;13:e19702. doi: 10.7717/peerj.19702 (PMC12278940; doi:10.7717/peerj.19702)
Supplement: Supplemental Information 1 [file peerj-13-19702-s001.pdf]

The categorical variables in the raw dataset have the following meaning: 0 = absent and 1 = present.
